# Timing and Frequency of Surveillance After Resection of Extremity and Trunk Soft Tissue Sarcoma: Identifying Opportunities for Improvement

**DOI:** 10.1245/s10434-026-19337-2

**Published:** 2026-03-10

**Authors:** Neha Malik, Raymond Traweek, Heather G. Lyu, Russell Witt, Yi-Ju Chiang, Samuel Cass, Brandon Cope, Alexander Mericli, Heather A. Lillemoe, Christopher Scally, Keila Torres, Kelly Hunt, Christina L. Roland, Emily Z. Keung

**Affiliations:** 1 Department of Surgical Oncology, The University of Texas MD Anderson Cancer Center, Houston, TX USA; 2 Department of Surgical Oncology, UVAHealth, Charlottesville, VA USA; 3 Department of Head and Neck Surgery, The University of Texas MD Anderson Cancer Center, Houston, TX USA; 4 Department of Surgery, Keesler Medical Center, Biloxi, MS USA; 5 Department of Plastic Surgery, The University of Texas MD Anderson Cancer Center, Houston, TX USA

## Abstract

**Introduction:**

Surveillance practices after resection of extremity and truncal soft-tissue sarcoma (ETSTS) vary, with guidelines recommending physical exam and imaging every 3–6 months. Local and distant recurrence risks differ based on clinicopathological features including tumor grade and histopathologic subtype. The aim of this study was to evaluate surveillance patterns after ETSTS resection at our high-volume sarcoma referral center and determine if opportunities exist to de-escalate intensity of surveillance in appropriate patients.

**Methods:**

A retrospective review of patients with primary ETSTS who underwent resection and surveillance at our institution from 2016-2021 was performed. Patients with less than 2 years of follow-up after resection were excluded. Sarculator, a validated nomogram for soft-tissue sarcoma, was used to stratify patients into high- (< 60%) and low-risk (≥ 60%) prognostic groups based on their predicted overall survival (OS) at 10 years.

**Results:**

The cohort of 296 patients included 112 high- and 184 low-risk. 5-year OS was 70.5% in the high-risk group versus 90.9% in the low-risk group (*P* < 0.001). Most patients did not recur during the study period (*n* = 206). 26 patients recurred within 6 months from their initial surgery (18 high-risk, 8 low-risk) and all were offered immediate treatment after recurrence diagnosis. Of this cohort, 84.6% were offered immediate treatment after diagnosis of recurrence.

**Conclusion:**

Surveillance strategies after primary resection should be tailored to patient’s individual risk. Patients with low-risk clinicopathologic features have a lower chance of recurrence in the first two years and frequent surveillance visits may not impact their oncologic outcomes.

**Supplementary Information:**

The online version contains supplementary material available at 10.1245/s10434-026-19337-2.

The extremities and trunk are the most common site of soft tissue sarcoma (STS). With the introduction of new therapies, the 2-year disease-free survival of patients with primary extremity STS (ETSTS) has been reported to reach 67%.^1^ Local recurrence is not common in ETSTS, occurring in only less than 15% of patients who underwent multimodality therapy with margin-negative surgery and radiation therapy.^2^ Timely diagnosis can result in successful treatment of a recurrence and a recurrence-free survival (RFS) of up to 72% at 5 years.^3^

Local and distant recurrence risks in ETSTS differ based on clinicopathologic features, including tumor grade and histopathologic subtype, and patients who present with lower-risk disease may not require the same level of surveillance as those who present with higher-risk disease. Although clinicians likely tailor surveillance strategy informed by a patient’s perceived risk of recurrence, most guidelines and recommendations regarding surveillance after completion of ETSTS treatment are relatively nonspecific and broad. The current National Comprehensive Cancer Network guidelines recommend that patients undergo surveillance visits with a medical history and physical examination every 3 to 6 months for the first 2 to 3 years after completion of treatment. Imaging is recommended depending on the risk of recurrence, but may not be required for areas easily followed by physical examination.^4^

The European Society of Medical Oncology guidelines state that patients with high-grade disease should be followed every 3 to 4 months and those with low-grade disease every 6 months.^5^ However, the guidelines do not include a clear definition of what constitutes high- or low-risk disease. Thus, there is significant variability in how providers choose to follow up with their patients in clinical practice. Multiple survey-based studies have shown differences in surveillance practices among providers. A survey conducted among members of the Society of Surgical Oncology showed that surveillance practices varied widely among members.^6^ A similar study by the Musculoskeletal Tumor Society showed disagreement in surveillance guidelines for patients with low-grade sarcomas.^7^

We previously examined the surveillance practices for patients after resection of primary retroperitoneal liposarcoma at our institution and identified potential opportunities to de-escalate the intensity of surveillance without impacting outcomes for a subset of patients.^8^ We found that patients who had undergone surgery for retroperitoneal well-differentiated liposarcoma (WDLPS) could be followed at 6-month intervals rather than 3- or 4-month intervals with no impact on treatment decisions or delay in initiation of treatment for any recurrent disease. However, the biology of retroperitoneal WDLPS is variable and has a different prognosis than some subtypes of STS. Additionally, the management of recurrent STS in the retroperitoneum/abdomen may differ from management of recurrent ETSTS, including active surveillance as an initial treatment strategy. Thus, although de-escalation of surveillance intensity may be a reasonable option for patients after resection for primary retroperitoneal WDLPS with respect to decreasing “scanxiety,” health care resource utilization, and other considerations, for example, this may not hold true for patients with ETSTS.

Furthermore, the optimal surveillance strategies for patients with ETSTS will likely differ based on patient and tumor characteristics, and the perceived risk of local recurrence and distant metastasis. Callegaro et al.^9^ developed and validated a prognostic nomogram, Sarculator, in a cohort of Italian sarcoma patients and found that it was able to accurately predict overall survival (OS) and distant metastasis rates for adults with resected ETSTS. Sarculator has been further validated in patients with primary ETSTS within the United States and can be used by providers to better predict patients’ OS duration on the basis of age, tumor size, tumor grade, and histology.^10^ Although Sarculator has been validated for predicting OS and the incidence of distant metastasis, no studies have evaluated use of patients’ Sarculator values to better refine their postoperative surveillance strategy.

This study aimed to evaluate surveillance practices for ETSTS at our institution and identify potential opportunities for de-escalation of postoperative surveillance intensity based on patients’ tumor features and Sarculator-predicted 10-year OS rate.

## Methods

We performed a retrospective review of all adults (age ≥ 18 years) with primary ETSTS who underwent surgical resection at The University of Texas MD Anderson Cancer Center between January 2016 and September 2021 and received at least 2 years of follow-up care and surveillance at MD Anderson. All the patients were identified through review of electronic medical records. This study was approved by the Institutional Review Board of MD Anderson.

Because the COVID-19 pandemic occurred during the study period and many patients opted not to travel for follow-up visits, we also included patients who received surveillance imaging outside MD Anderson that was interpreted by the radiology department at MD Anderson. The study excluded patients who were already receiving treatment for another primary neoplasm at the time of their ETSTS diagnosis.

The electronic medical records of all the patients were reviewed. The variables collected included the following patient demographics: diagnosis method, tumor characteristics (e.g., primary tumor location, size, and grade), treatments received, surveillance imaging methods used to monitor for local and distant recurrence, surveillance imaging dates, and imaging results at each surveillance visit. For patients whose disease recurred, the time to recurrence was calculated from the date of primary ETSTS resection to the date of diagnosis of recurrence. The treatment strategy used for recurrent disease, such as chemotherapy, radiation therapy, or surgery, also was documented, together with the dates treatment was initiated for recurrence.

The outcomes of interest included RFS and OS durations. Duration of RFS was calculated from the date of primary ETSTS resection to the date recurrence was diagnosed or the date of last the follow-up visit if the patient did not experience recurrence. Recurrence diagnosis was defined using imaging findings. Duration of OS was calculated from the date of primary ETSTS diagnosis to the date of death or last follow-up visit.

Predicted 5- and 10-year OS and disease-free survival rates were calculated using Sarculator, a validated prognostic nomogram. The patients then were stratified into two groups based on a well-established cutoff at a predicted 10-year OS rate of 60%. This cutoff was defined by Pasquali et al.^11^ using the results from EORTC-STBSG 62931, a randomized controlled trial that analyzed the impact of adjuvant chemotherapy versus observation. They found that adjuvant chemotherapy was associated with improved oncologic outcomes for patients with a predicted 10-year OS rate lower than 60%, hereafter referred to as “high risk.” No benefit was observed among those with a predicted 10-year OS rate higher than 60%, referred to as “low risk.”^11^ Descriptive statistics were used to show the distribution of variables across the overall patient cohort, the high- and low-risk groups, and the cohort of patients who experienced early recurrence.

A chi-square analysis was used to compare categorical variables, and Student’s *t* test was used for continuous variables. Uni- and multivariable Cox regression analyses were used to determine the association between preoperative clinicopathologic factors and survival. Kaplan-Meier survival plots for RFS and OS rates were constructed to compare patients with high- and low-risk disease and patients who experienced early disease recurrence with all other patients. Statistical significance was defined by a *P* value lower than 0.05.

## Results

### Patient Demographics

Between 2016 and 2021, 296 patients underwent ETSTS resection and had at least 2 years of surveillance follow-up evaluation at MD Anderson, with a median follow-up period of 3.71 years (range, 0.1–9.8 years) (Table 1). Of these 296 patients, 184 were classified as low risk (Sarculator 10-year OS rate ≥60%) and 112 as high-risk (Sarculator 10-year OS rate <60%). The median age at diagnosis was 54 years (interquartile range [IQR], 42.0–66.0 years) in the low-risk group (*P* < 0.001) and 67.5 (IQR, 57.5–74.0 years) in the high-risk group (*P* < 0.001). The two groups did not differ in terms of gender distribution (males 50%, females 50%), and most of the patients identified as white or Caucasian (81.8%).

**Table 1:** Demographics and clinical characteristics

	Overall cohort (*n* =296) *n* (%)	High risk (*n* =112) *n* (%)	Low risk (*n* =184) *n* (%)	*P* Value
*Median age at diagnosis: years* (IQR)	60 (47–69)	67.5 (57.5–74)	54 (42–66)	<0.001
*Gender*				0.632
Male	148 (50)	54 (48.2)	94 (51.1)	
Female	148 (50)	58 (51.8)	90 (48.9)	
*Race*				0.213
White or Caucasian	242 (81.8)	92 (82.1)	150 (81.5)	
Black or African American	20 (6.8)	10 (8.9)	10 (5.4)	
Asian	10 (3.4)	4 (3.6)	6 (3.3)	
Other	23 (7.8)	5 (4.5)	18 (9.8)	
Unknown	1 (0.3)	1 (0.9)	0 (0)	
*Histology*				0.066
UPS	89 (30.1)	33 (29.5)	56 (30.4)	
Other	86 (29.1)	35 (31.3)	51 (27.7)	
Myxofibrosarcoma	33 (11.2)	9 (8.0)	24 (13.0)	
LMS	24 (8.1)	6 (5.4)	18 (9.8)	
Myxoid LPS	23 (7.8)	0 (0)	23 (12.5)	
DD/pleomorphic LPS	16 (5.4)	12 (10.7)	4 (2.2)	
Synovial	15 (5.1)	9 (8.0)	6 (3.3)	
Vascular	7 (2.4)	6 (5.4)	1 (0.5)	
MPNST	3 (1.0)	2 (1.8)	1 (0.5)	
*Median tumor size: cm (IQR)*	8.6 (5–12.6)	12.3 (9.7–17.1)	5.8 (3.5–9)	<0.001
*Tumor grade*				<0.001
*Diagnosis method*				0.254
Excisional biopsy	168 (56.8)	57 (50.9)	111 (60.3)	
Core needle biopsy	77 (26.0)	37 (33.0)	40 (21.7)	
Shave biopsy	7 (2.4)	2 (1.8)	5 (2.7)	
Punch biopsy	6 (2.0)	1 (0.9)	5 (2.7)	
FNA	1 (0.3)	0 (0)	1 (0.5)	
Unknown	37 (12.5)	15 (13.4)	22 (12.0)	
*Resection type*				0.029
R0/R1	295 (99.7)	112 (100)	183 (99.5)	
R2	1 (0.3)	0 (0)	1 (0.5)	
*Chemotherapy*				0.719
Neoadjuvant	85 (28.7)	36 (32.1)	49 (26.6)	
Adjuvant	10 (3.4)	4 (3.6)	6 (3.3)	
Combination	7 (2.4)	3 (2.7)	4 (2.2)	
None	194 (65.5)	69 (61.6)	125 (67.9)	
*Radiation*				0.233
Preoperative	243 (82.1)	97 (86.6)	146 (79.4)	
Postoperative	5 (1.7)	2 (1.8)	3 (1.6)	
None	48 (16.2)	13 (11.6)	35 (19.0)	
*Initial surveillance intervals*				0.603
Every 3 months	253 (85.5)	97 (86.6)	156 (84.8)	
Every 4 months	27 (9.1)	7 (6.3)	20 (10.9)	
Every 6 months	6 (2.0)	2 (1.8)	4 (2.2)	
Every 12 months	11 (0.35)	0 (0)	1 (0.5)	
Unknown	9 (3.0)	6 (5.4)	3 (1.6)	

IQR, interquartile range; UPS, undifferentiated pleomorphic sarcoma; LMS, leiomyosarcoma; LPS, liposarcoma; DD, dedifferentiated; MPNST, malignant peripheral nerve sheath tumor; FNA, fine-needle aspiration

### Tumor Characteristics

Across the entire cohort, the median tumor size was 8.6 cm. The low-risk group had smaller tumors, with a median size of 5.8 cm compared with 12.3 cm in the high-risk group (*P* < 0.001). Tumor grade also varied significantly between the two groups. Most of the patients presented with high-grade disease (70.3%), but only 58.2% of the low-risk patients presented with high-grade disease compared with 90.2% of the high-risk patients (*P* < 0.001). Most of the patients (82.1%) received preoperative radiation therapy, and 34.5% received some form of chemotherapy, with 31.1% receiving neoadjuvant chemotherapy (Table 1). Receipt of preoperative radiation therapy or chemotherapy did not statistically differ significantly between the low- and high-risk groups.

### Patterns of Surveillance and Patient Outcomes

Most of the patients underwent surveillance imaging every 3 (85.5%) or 4 (9.1%) months and remained disease-free after ETSTS resection (Table 1). Initial surveillance imaging frequency did not differ significantly between the high- and low-risk groups (*P* = 0.603). The median time to recurrence for the entire cohort was 12.7 months (IQR, 4.7–29.5 months) (Table 2). Although time to recurrence did not statistically differ significantly between the high- and low-risk groups, the median time to recurrence in the low-risk group was almost twice that in the high-risk group (1.56 vs 0.89 years; *P* = 0.086). In addition, the patients with high-risk sarcoma were much more likely to experience recurrence in 3 months (*n* = 9, 8.0%) than those with low-risk disease (*n* = 1; 0.5%; *P* = 0.018). For 26 patients (8.8%), recurrence occurred within 6 months after surgery (local recurrences only for 3 patients, distant recurrences only for 20 patients, and both for 3 patients). Of the 26 patients, 18 were classified as high risk and 8 as low risk. For 64 patients (21.6%), ETSTS recurred 6 months after surgery (local recurrences only for 31 patients [48.4%], distant recurrences only for 31 patients [50.8%], and both for 2 patients [3.3%]).

**Table 2:** Patient outcomes

	Overall cohort (*n* = 296) *n* (%)	High risk (*n* = 112) *n* (%)	Low risk (*n* = 184) *n* (%)	*P* value
*Recurrence*				0.251
Any recurrence	90 (30.4%)	48 (42.9%)	42 (22.8%)	
Local recurrence	34 (11.5%)	15 (13.4%)	19 (10.3%)	
Distant recurrence	51 (17.2%)	30 (26.8%)	21 (11.4%)	
Distant & local recurrence	5 (1.7%)	3 (2.7%)	2 (1.1%)	
*Recurrence timing*^*a*^				
Median time to recurrence: months (IQR)	12.7 (4.7–29.5)	10.7 (3.7–30.1)	18.7 (12.3–29.1)	0.086
Recurrence in <3 months	10 (3.4%)	9 (8.0%)	1 (0.5%)	0.018
Recurrence in <4 months	17 (5.7%)	12 (10.7%)	5 (2.7%)	0.177
Recurrence in <6 months	26 (8.8%)	18 (16.1%)	8 (4.4%)	0.065
Recurrence in <12 Months	44 (14.9%)	29 (25.9%)	15 (8.15%)	0.022
*Survival*				
5-Year RFS	64.9	51.2	72.2	<0.001
5-Year OS	83.2	70.5	90.9	<0.001
Sarculator predicted 10-year OS (months)	68	45	78	<0.001

IQR, interquartile range; RFS, recurrence-free survival; OS, overall survival

^a^Recurrence timing is cumulative. Patients who recurred in less than 3 months are also counted in the less than 4 month group etc

When the patients who had recurrence in less than 6 months were compared with those who had recurrence in more than 6 months, the only significant difference was tumor size. The median tumor size in the patients who experienced recurrence within 6 months of ETSTS resection was 16 cm compared to 7.65 cm in those who experienced recurrence longer than 6 months after surgery (*P* < 0.001). There were otherwise no significant differences in patients’ demographics, tumor grade, diagnosis, or treatment. There also were no statistically significant differences in the initial patterns of surveillance between the patients who experienced recurrence within 6 months, those who experienced recurrence after 6 months, and those who never experienced recurrence (*P* = 0.818; Table S1).

**Fig. 1:**
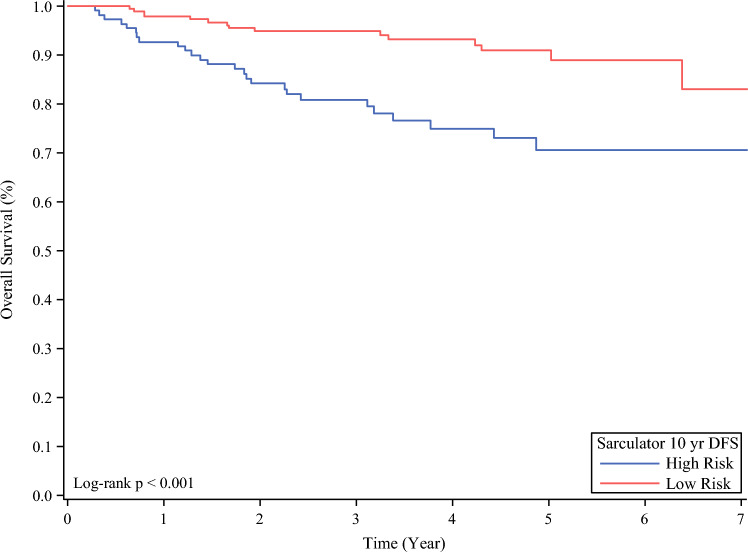
Overall survival by group

The 5-year RFS was 51.2% in the high-risk group and 72.2% in the low-risk group (*P* < 0.001). The 5-year OS rate was 70.5% in the high-risk group versus 90.9% in the low-risk group (*P* < 0.001; Fig. 1). For the patients who experienced recurrence in less than 6 months, the 5-year OS was 0% versus 69.9% for those who experienced recurrence in ≥6 months. When the 5-year OS of our entire cohort was compared with the Sarculator-predicted 5-year OS, our patient population had superior survival outcomes (83.2% vs 77.5%). However, we found that Sarculator was not as accurate in predicting the 5-year OS for the patients who experienced early recurrence (Sarculator [54.4%] vs actual [0%]; (Table S2).

### Impact of Postoperative Surveillance Intervals on Management of Recurrence

Within 6 months after undergoing primary ETSTS resection, 26 patients (8.8%) experienced recurrence. Of these patients, 24 had recurrent or metastatic disease diagnosed at the time of their scheduled surveillance imaging (distant recurrence only [*n* = 20], local recurrence only [*n* = 3], and both local and distant recurrence [*n* = 3]). In one patient, local recurrence was diagnosed after a tissue biopsy during a plastic surgery procedure showed recurrent disease, and another patient had symptoms related to metastatic disease to the spine. Of the 26 patients, 18 had high-risk disease and 8 had low-risk disease. Of patients who had recurrence within 6 months, 81.5% had an R0 resection during their initial surgery, and margin status did not differ between the high- and low-risk cohorts (*P* = 0.628). We evaluated how recurrences were managed in this cohort and found that the majority of the patients (84.6%) underwent immediate treatment after the diagnosis of recurrence. Of the four patients who did not receive immediate treatment, two elected to transition to hospice, and two did not follow up at our institution after diagnosis of their recurrence. Of the 26 patients who did undergo treatment, 16 (61.5%) were treated with chemotherapy, 1 (3.9%) was treated with radiation therapy, 3 (11.5%) underwent surgery, and 2 (7.7%) received other forms of treatment (immunotherapy and tazemetostat). For the 22 patients who received immediate treatment after a diagnosis of recurrence, changing their surveillance frequency from every 3 months to every 4 or 6 months would have had a direct impact on the management of their recurrence, resulting in a delay in treatment initiation.

## Discussion

In our cohort of 296 ETSTS patients who underwent both resection and surveillance at our institution, we found that the timing of surveillance had a substantial effect on treatment planning for recurrences. The majority of our patient cohort had an initial scheduled surveillance strategy with imaging every 3 months. When evaluating the potential impact of more frequent surveillance visits, we found that extending the length of time between surveillance intervals would have affected the timing of treatment initiation for the 22 patients (7.4%) whose disease recurred within 6 months after primary ETSTS resection and who underwent immediate treatment after recurrence diagnosis. Although the characteristics of the patients who experienced recurrence within 6 months after initial surgery did not differ significantly from those of the patients who experienced recurrence after 6 months, their median tumor size was twice that of those who experienced recurrence later (16 vs 7.35 cm).

We found no association between tumor grade and early recurrence, in contrast to Tirotta et al.^13^ who identified higher tumor grade as the only significant independent predictor of early recurrence among patients treated for primary retroperitoneal sarcoma. Our study analyzed only patients with ETSTS, whereas their study investigated patients with primary RPS, which may explain this difference in our findings. Additionally, although we did not find any statistically significant difference in tumor grade between patients who experienced recurrence early versus late, we did observe a higher percentage of patients with high-grade disease in the early recurrence group versus the late recurrence group (88.5 vs 75%). This finding suggests that for patients with higher-risk features, earlier diagnosis of disease recurrence during more intensive surveillance at 3-month intervals results in earlier initiation of treatment of recurrent disease.

However, whether earlier treatment initiation for recurrent ETSTS is associated with changes in OS remains unknown. In addition to using the Sarculator-predicted OS rate to define patients as high or low risk, we compared our cohort’s OS rate with the predicted Sarculator OS rate. We found that overall, our patients had slightly better OS rates than those predicted by Sarculator. However, when evaluating our cohort of patients who experienced recurrence within 6 months after their initial surgery, Sarculator tended to overestimate their OS rate. This suggests that although more frequent surveillance may aid in identifying and initiating treatment for recurrences earlier, this may not impact patients’ long-term oncologic outcomes.

Previous study from our institution of the retroperitoneal liposarcoma population has shown that longer surveillance intervals may not affect patients’ treatment course or OS rate.^8^ In this cohort, surgery for recurrence was performed a median of 6.1 months after the detection of a WDLPS recurrence. However, this was not the case in our population of patients with primary ETSTS after resection who experienced early recurrence. Treatment was initiated almost immediately after the detection of a recurrence for the patients with a history of resected primary ETSTS, possibly due to the greater ease and accessibility of local recurrences, the higher potential metastatic risk, and the availability of more effective systemic therapies for some ETSTS histologies compared with intra-abdominal and/or retroperitoneal WDLPS recurrences. Most of the patients in the current cohort who experienced early recurrence were offered immediate treatment, with chemotherapy as the most common method. Surveillance guidelines currently do not differentiate between the location of the primary tumor and recommend surveillance visits only every 3 to 6 months. Our research suggests that surveillance strategies could be tailored to the primary tumor location.

In addition to tumor location, patient risk based on the Sarculator 10-year OS rate may be beneficial for determining whether patients will benefit from surveillance visits every 3 months versus every 4 to 6 months. Sarculator currently uses patient age, tumor size, tumor grade, and histology to calculate DFS and OS rates. In our patient population, we found that only tumor size was statistically significant in differentiating between patients who experienced recurrence early, late, or not at all. Voss et al.^10^ found that although Sarculator was an accurate predictor of OS rate for patients with ETSTS, it did provide more accurate predictions in certain patient populations, which may explain some of the discrepancy between our patient survival outcomes and those predicted by Sarculator. Although our cohort had overall better outcomes than what Sarculator predicted, it can still provide useful risk-related data for physicians and patients. In addition, as Sarculator continues to integrate new features into its algorithm, the risk predictions will continue to improve. Using the Sarculator 10-year OS rate to stratify patients into high and low risk may provide patients with a more tailored approach to surveillance. In our cohort, the low-risk patients statistically had a significantly lower risk of recurrence, but the majority still were undergoing surveillance visits every 3 months. Because of this study period and partly as a result of this work, our team currently is routinely using Sarculator for risk stratification of patients with primary ETSTS and is identifying opportunities to develop, study, and implement strategies for de-escalation of surveillance for “low-risk” patients.

There is limited evidence for evaluating how surveillance contributes to ETSTS patients’ anxiety and quality of life. However, research in other cancer populations has shown that “scanxiety” can result in a poorer quality of life for cancer patients.^12^ Further studies are needed to investigate the impact of less frequent surveillance visits on patients’ anxiety and quality of life. Studies also should evaluate the costs of surveillance to both patients and the health care system.

Our study had several limitations. The first was our retrospective design, which may have introduced sources of bias and limited a full understanding of the rationale providers and patients used to guide their decisions regarding treatment of ETSTS recurrences in this study. Another limitation was our small sample size, which may mean the study was underpowered to detect differences between our groups. Despite the small sample size, our study represents the only work to date that used Sarculator to stratify patients into high- and low-risk groups to assess surveillance strategies after primary ETSTS treatment.

In conclusion, we found that for patients who underwent resection of ETSTS, the timing of surveillance visits impacted the course of treatment, especially for patients defined as high risk by Sarculator. Unlike the patients with retroperitoneal WDLPS, the patients with ETSTS were more likely to undergo treatment immediately after diagnosis of a recurrence However, the impact that earlier detection of disease recurrence in high- versus low-risk patients as defined by Sarculator had on patients’ oncologic outcomes remains unclear. Although there is no “one-size-fits-all” approach for the surveillance of patients with ETSTS, tools such as Sarculator can guide providers and patients toward adoption of surveillance approaches tailored to and informed by patients’ recurrence risks.

## Supplementary Information

Below is the link to the electronic supplementary material.

Supplementary file1 (DOCX 13 KB)

## References

[1] Mowery YM, Ballman KV, Hong AM, et al. Safety and efficacy of pembrolizumab, radiation therapy, and surgery versus radiation therapy and surgery for stage III soft tissue sarcoma of the extremity (SU2C-SARC032): an open-label, randomised clinical trial. *The Lancet*. 2024;404(10467):2053–64. 10.1016/S0140-6736(24)01812-9. 10.1016/S0140-6736(24)01812-9 PMC11842127 39547252

[2] O’Sullivan B, Davis AM, Turcotte R, et al. Preoperative versus postoperative radiotherapy in soft-tissue sarcoma of the limbs: a randomised trial. *The Lancet*. 2002;359(9325):2235–41. 10.1016/S0140-6736(02)09292-9. 10.1016/S0140-6736(02)09292-9 12103287

[3] Rosenberg SA, Tepper JO, Glatstein EL, et al. The treatment of Soft-tissue sarcomas of the extremities: prospective randomized evaluations of (1) Limb-sparing surgery plus radiation therapy compared with compared with amputation and (2) the role of adjuvant chemotherapy. *Ann Surg*. 1982;196(3):305–15. 10.1097/00000658-198209000-00009. 7114936 10.1097/00000658-198209000-00009 PMC1352604

[4] National Comprehensive Cancer Network. *Soft Tissue Sarcoma.*; Retrieved 2024 at https://www.nccn.org/professionals/physician_gls/pdf/sarcoma.pdf.

[5] Gronchi A, Miah AB, Dei Tos AP, et al. Soft tissue and visceral sarcomas: ESMO–EURACAN–GENTURIS clinical practice guidelines for diagnosis, treatment and follow-up. *Ann Oncol*. 2021;32(11):1348–65. 10.1016/j.annonc.2021.07.006. 34303806 10.1016/j.annonc.2021.07.006

[6] Johnson FE, Sakata K, Kraybill WG, Gibbs JF, Beitler AL, Sarkar S, Audisio RA, Virgo KS. Long-term management of patients after potentially curative treatment of extremity soft tissue sarcoma: practice patterns of members of the Society of Surgical Oncology. *Surg Oncol*. 2005;14(1):33–40. 10.1016/j.suronc.2004.12.001. 15777888 10.1016/j.suronc.2004.12.001

[7] Ries Z, Gibbs CP Jr, Scarborough MT, Miller BJ. Pulmonary surveillance strategies following sarcoma excision vary among orthopedic oncologists: a survey of the Musculoskeletal Tumor Society. *Iowa Orthop J*. 2016;36:109–16. 27528846 PMC4910805

[8] Keung EZ, Rajkot N, Torres KE, et al. Evaluating the impact of surveillance follow-up intervals in patients following resection of primary well-differentiated liposarcoma of the retroperitoneum. *Ann Surg Oncol*. 2021;28(1):570–5. 10.1245/s10434-020-08582-8. 32409969 10.1245/s10434-020-08582-8 PMC7666053

[9] Callegaro D, Miceli R, Bonvalot S, et al. Development and external validation of two nomograms to predict overall survival and occurrence of distant metastases in adults after surgical resection of localised soft-tissue sarcomas of the extremities: a retrospective analysis. *Lancet Oncol*. 2016;17(5):671–80. 10.1016/S1470-2045(16)00010-3. 27068860 10.1016/S1470-2045(16)00010-3

[10] Voss RK, Callegaro D, Chiang YJ, et al. Sarculator is a good model to predict survival in resected extremity and trunk sarcomas in US patients. *Ann Surg Oncol*. 2022;29:4376–85. 10.1245/s10434-022-11442-2. 10.1245/s10434-022-11442-2 35224688

[11] Pasquali S, Pizzamiglio S, Touati N, et al. The impact of chemotherapy on survival of patients with extremity and trunk wall soft tissue sarcoma: revisiting the results of the EORTC-STBSG 62931 randomised trial. *Eur J Cancer*. 2019;109:51–60. 10.1016/j.ejca.2018.12.009. 30690293 10.1016/j.ejca.2018.12.009

[12] Derry-Vick HM, Heathcote LC, Glesby N, et al. Scanxiety among adults with cancer: a scoping review to guide research and interventions. *Cancers*. 2023;15:1381. 10.3390/cancers15051381. 36900174 10.3390/cancers15051381 PMC10000102

[13] Tirotta F, Fadel MG, Baia M, et al. Risk factors for the development of early recurrence in patients with primary retroperitoneal sarcoma. *Ann Surg Oncol*. 2023;30:6875–83. 10.1245/s10434-023-13754-3. 37423926 10.1245/s10434-023-13754-3

