# Supplementary material for: Timing and Frequency of Surveillance After Resection of Extremity and Trunk Soft Tissue Sarcoma: Identifying Opportunities for Improvement
Source: Ann Surg Oncol. 2026 Mar 10;33(7):6859–66. doi: 10.1245/s10434-026-19337-2 (PMC13242450; doi:10.1245/s10434-026-19337-2)
Supplement: Supplementary file 1 — Supplementary file1 (DOCX 13 KB) [file 10434_2026_19337_MOESM1_ESM.docx]

**Supplemental Table 1: Demographics and Clinical Characteristics by Recurrence Timing**

| **(n, %)** | **Recurrence ≥ 6 months**  **(n =64)** | **Recurrence < 6 months**  **(n =26)** | **p value** |
| --- | --- | --- | --- |
| **Age at Diagnosis, median (IQR)** | 61.5 (52-72.5) | 67.5 (59-73) | 0.135 |
| **Gender** |  |  | 0.999 |
| Male | 32 (50%) | 13 (50%) |  |
| Female | 32 (50%) | 13 (50%) |  |
| **Race** |  |  | 0.769 |
| White or Caucasian | 50 (78.1%) | 22 (84.6%) |  |
| Black or African American | 5 (7.8%) | 1 (3.9%) |  |
| Asian | 3 (4.7%) | 0 (0%) |  |
| Other | 6 (9.4%) | 3 (11.5%) |  |
| Unknown | 0 (0%) | 0 (0%) |  |
| **Histology** |  |  | 0.278 |
| UPS | 18 (28.3%) | 12 (46.2%) |  |
| Other | 19 (29.7%) | 3 (11.5%) |  |
| Myxofibrosarcoma | 9 (14.1%) | 2 (7.7%) |  |
| LMS | 4 (6.3%) | 0 (0%) |  |
| Myxoid LPS | 3 (4.7%) | 2 (7.7%) |  |
| DD/Pleomorphic LPS | 5 (7.8%) | 4 (15.4%) |  |
| Synovial | 3 (4.7%) | 1 (3.9%) |  |
| Vascular | 2 (3.1%) | 2 (7.7%) |  |
| MPNST | 1 (1.6%) | 0 (0%) |  |
| **Tumor Size (cm), median (IQR)** | 7.7 (4.7-13.3) | 16 (10-21) | <0.001 |
| **Tumor Grade** |  |  | 0.189 |
| Low | 8 (12.5%) | 0 (0%) |  |
| Intermediate | 8 (12.5%) | 3 (11.5%) |  |
| High | 48 (75%) | 23 (88.5%) |  |

**Supplemental Table 2: Actual 5-year OS Compared to Sarculator Predicted 5-year OS**

| **(n, %)** | **Actual 5-year OS** | **Sarculator Predicted 5-year OS** |
| --- | --- | --- |
| **Whole Cohort  (n=296)** | 83.2 | 73.1 |
| **High-Risk (n=112)** | 70.5 | 54.2 |
| **Low-Risk (n=184)** | 90.9 | 84.6 |
| **Recurrence Timing** |  |  |
| ≥ 6 months (n=64) | 69.9 | 70.6 |
| < 6 months | 0 | 54.4 |
| No Recurrence | 97.5 | 76.3 |
